# Astrocyte-Neuron Metabolic Crosstalk in Neurodegeneration: A Mitochondrial Perspective

**DOI:** 10.3389/fendo.2021.668517

**Published:** 2021-05-07

**Authors:** Patrycja Mulica, Anne Grünewald, Sandro L. Pereira

**Affiliations:** ^1^ Luxembourg Centre for Systems Biomedicine, University of Luxembourg, Esch-sur-Alzette, Luxembourg; ^2^ Institute of Neurogenetics, University of Lübeck, Lübeck, Germany

**Keywords:** metabolism, astrocytes, neurons, neurodegeneration, Parkinson’s disease, Alzheimer’s disease

## Abstract

Converging evidence made clear that declining brain energetics contribute to aging and are implicated in the initiation and progression of neurodegenerative disorders such as Alzheimer’s and Parkinson’s disease. Indeed, both pathologies involve instances of hypometabolism of glucose and oxygen in the brain causing mitochondrial dysfunction, energetic failure and oxidative stress. Importantly, recent evidence suggests that astrocytes, which play a key role in supporting neuronal function and metabolism, might contribute to the development of neurodegenerative diseases. Therefore, exploring how the neuro-supportive role of astrocytes may be impaired in the context of these disorders has great therapeutic potential. In the following, we will discuss some of the so far identified features underlining the astrocyte-neuron metabolic crosstalk. Thereby, special focus will be given to the role of mitochondria. Furthermore, we will report on recent advancements concerning iPSC-derived models used to unravel the metabolic contribution of astrocytes to neuronal demise. Finally, we discuss how mitochondrial dysfunction in astrocytes could contribute to inflammatory signaling in neurodegenerative diseases.

## Introduction

**Figure 1:**
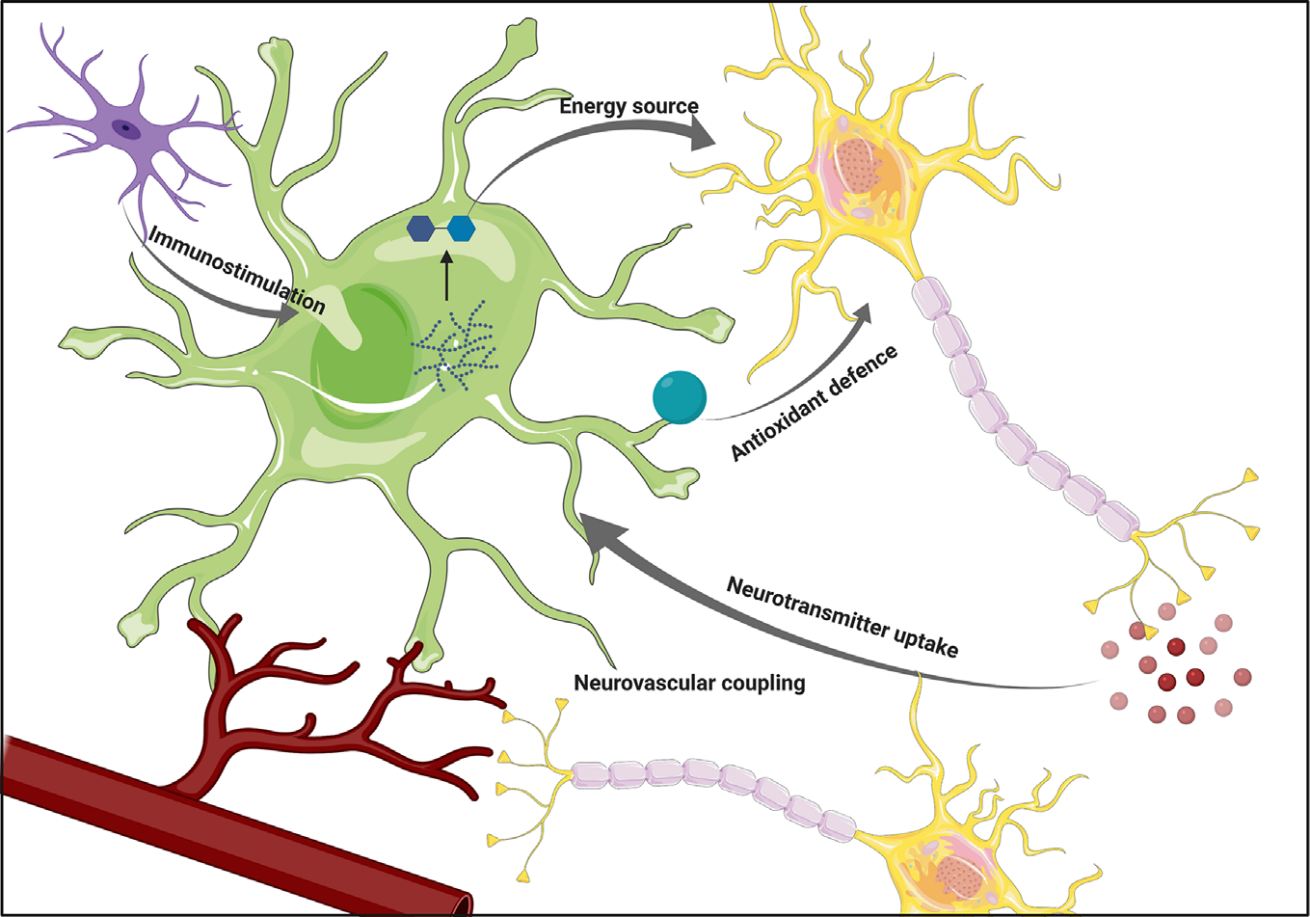
The overview of astrocytic functions. Astrocytes support neuronal functions by providing energy substrates and supporting antioxidant defense. Their ability to participate in the immune response by sensing cytokines secreted by microglia, as well as participation in neurovascular coupling positions them as a crucial component of the interplay between various brain cell types. Figure created with BioRender.com using images adapted from Servier Medical Art by Servier, licensed under a Creative Common Attribution 3.0 Unported License http://smart.servier.com/.

Neurodegenerative diseases can be grouped into a large class of disorders characterized by the gradual loss of various neuronal populations (1). The symptoms can be diverse depending on the cell types being affected, ranging from dementia and motor dysfunction to behavioral alterations (2). The incidence of Alzheimer’s (AD) and Parkinson’s disease (PD) was estimated in 2016 to reach globally over 43 and 6 million cases respectively, which positions them among the most common neurodegenerative disorders to date (3). Both of these diseases are associated with an extensive accumulation of protein aggregates; in AD patient brains such abnormalities are formed mainly by amyloid-beta (Aβ) and Tau proteins, whereas α-synuclein (α-SYN) is linked to the pathology of PD (2). So far, neurodegenerative diseases have been regarded primarily as neuronal pathologies, however, recent findings suggest that glial cells might play an important role in the disease formation and progression (4). In particular, astrocytes, as the cells supporting neuronal function, seem to be of great importance for our understanding of underlying disease mechanisms (5). In our review, we will focus on astrocytic metabolism as a key aspect of the astrocyte-neuron interplay (**Figure 1**), which, when disturbed, likely accelerates neuronal demise. In this context, we will illuminate recent advances concerning the role of mitochondria - organelles that are not only crucial for cellular metabolism, but also involved in inflammatory signaling. In addition, to highlight the need for research on neurodegeneration at the endogenous level, we compare the current literature on iPSC models used to study astrocytic metabolism.

## Heterogeneity of Astrocytes

Astrocytes represent the most abundant population among glial cells residing in the human brain (6). The classification of this highly heterogeneous group poses a major challenge, given the limited number of studies exploring the variability of astrocytes. Based on neuroanatomical studies and analysis of their morphology, astrocytes have been traditionally subdivided into four distinct classes such as (i) interlaminar, (ii) varicose projection, (iii) protoplasmic and (iv) fibrous. Interlaminar astrocytes are located in the layer I of the human cortex and are characterized by a round cell body and, in general, a short length of their processes. Nevertheless, they also possess a few processes, which expand to layers II-IV of the cortex but the function of those, as well as of interlaminar astrocytes on the whole, remains elusive. Despite being more uncommon, varicose projection astroglia, found in layers V-VI, attract attention due to the fact that they are specific to humans and higher-order primates. They typically develop short spiny processes together with one to five longer ones, which extend to the deep layers of the cortex and might terminate in the neuropil or on the blood vessels (7). Similarly to interlaminar astrocytes, the role played by varicose projection astrocytes is not yet fully understood. However, it was hypothesized that both of these subtypes might be involved in long-distance communication within the cortex (7). Layers II to VI of the cerebral cortex are populated by the most abundant group of astroglia, namely the protoplasmic astrocytes. They form numerous processes, which are homogeneously distributed and typically described as bulbous, creating overall a bushy morphology. Organized in distinct domains with minimum overlap between individual astrocytes, protoplasmic astroglia were suggested to influence neuronal activity in spatially and temporally coordinated units. Their involvement in metabolic support is also widely reported, similarly to their contribution to the regulation of blood flow (7, 8). The last group of astroglia, fibrous astrocytes, reside in the white matter and are typically larger in size, although containing less processes. As protoplasmic astrocytes, fibrous astroglia seem to be involved in the metabolic support but not in the modulation of neuronal activity due to the lack of synapses in the white matter (7, 9). Apart from the cells described above, other astroglia types were identified such as Bergmann glia in the cerebellum and Müller cells in the retina (10).

Most of the above-described neuroanatomical studies aiming at classifying astrocytic populations relied primarily on GFAP as a potent astrocytic marker - although its widespread application has recently been called into question. In the meantime, it became apparent that GFAP is not expressed in all mature and non-activated astrocytes in the central nervous system. Moreover, it was postulated that GFAP expression patterns might present regional differences (11). Accordingly, recent studies tried to overcome this limitation by using different markers such as Aldh1l1. Based on the observation that Aldh1l1 might serve as a general marker for astrocytes in the central nervous system (12), Lin et al. discovered five subpopulations of astrocytes in mouse brain, which were molecularly and functionally distinct, and suggested that similar types might be found in human brain. Interestingly, synaptogenesis was observed to be differently modulated by various identified subtypes suggesting functional heterogeneity of astrocytes (13). Recent developments in single-cell transcriptomics allowed identification of five to seven astrocytic subpopulations in the mouse nervous system, presenting various morphologies and functions (14, 15). This finding highlights that more research is needed to unravel the full spectrum of astrocytic heterogeneity.

## Cellular Functions of Astrocytes

Astrocytes are involved in a myriad of functions, mostly attributed to their supporting role for neurons. During development, they appear after completed neurogenesis and contribute to synapse pruning (16, 17). Besides their role in brain development, astrocytic processes extensively ensheath synapses and participate in neurotransmitter removal from the synaptic cleft, preventing excitotoxicity and direct contact of the neurotransmitter with neighboring synapses. In a similar fashion, astrocytes are pivotal for maintaining an ionic balance at the synapse, which is required for synaptic transmission (6). A strong body of evidence supports the idea of the ‘tripartite synapse’. Accordingly, astrocytes play not only a supportive role in synaptic functions, but also actively modulate neurotransmission. This hypothesis was supported by the observation that astrocytes can react to neuronal activity by increasing their intracellular Ca^2+^ levels, which in turn leads to the secretion of numerous gliotransmitters including glutamate, purines, GABA and D-serine (11). Such gliotransmitters are believed to participate in the feedback regulation of synaptic activities (18). Besides the perisynaptic processes that participate in tripartite synapses, astrocytes extend vascular processes (end-feet) that ensheath capillary endothelium and pericytes, giving rise to the blood-brain barrier (BBB), a selectively permeable structure that tightly controls the movement of molecules and cells between the vascular compartment and the brain parenchyma (19). This unique anatomic position, structurally mediating blood vessels and neurons, is influential for astrocytic roles in surveilling and buffering local environmental changes to nurture neurons with appropriate metabolites in a spatial-temporal manner, a process named neurovascular-coupling (NC), which will be discussed in more detail below (20). Furthermore, the recent discovery of the glymphatic system (21), a waste clearance system relying on perivascular tunnels defined by astrocytes, broadens the scope of astroglial involvement in CNS homeostasis. This system supports the elimination of byproducts of brain metabolism including neurotoxic proteins such as Aβ, countering their noxious accumulation in the brain parenchyma (22).

An important aspect of astrocytic physiology is their ability to respond to brain injury or CNS disease, a phenomenon known as reactive astrogliosis. The term collectively describes various molecular and cellular processes, which are highly heterogeneous and therefore challenging to summarize under a definitive classification. The response might range from mild astrogliosis, typically characterized by altered gene expression and occasional cell hypertrophy without astrocytic proliferation, to severe astrogliosis, which is accompanied by the increase of GFAP expression, cell hypertrophy and substantial proliferation. Pronounced reactive astrogliosis might lead to the formation of scar tissue, composed of proliferating astrocytes (23). Astrocytes also play an important role in neuroinflammation, since they can react to cytokines released by activated microglia (24) and secrete cytokines and chemokines (25) contributing to the ongoing inflammation process. This aspect is further discussed in section *Astrocytes and Mitochondria in Inflammation*.

## Astrocyte-Neuron Metabolic Crosstalk in Health and Neurodegenerative Diseases

**Figure 2:**
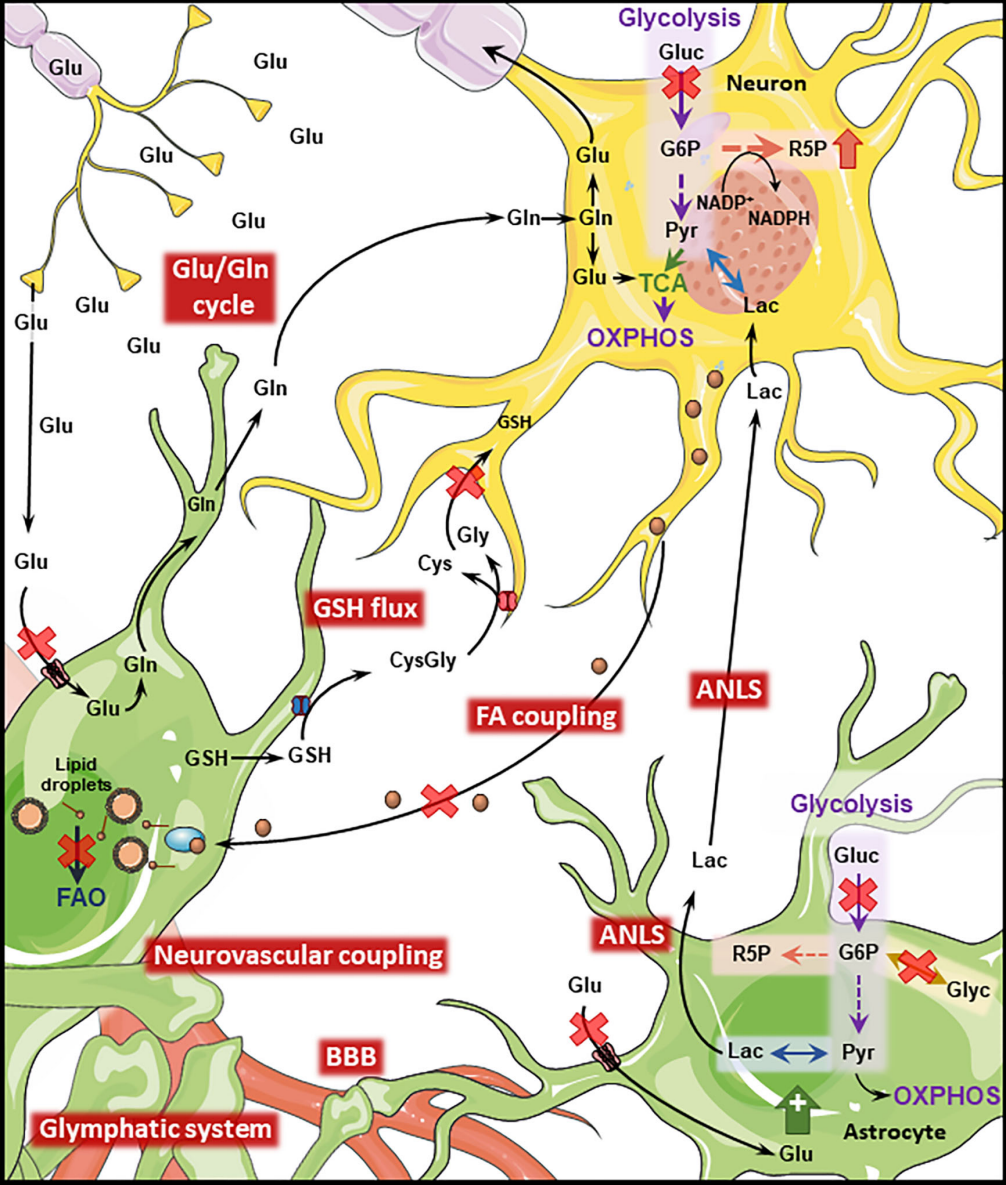
Disrupted Astrocyte-Neuron metabolic interplay in PD and AD. Metabolic interaction between astrocytes and neurons is disrupted in AD and PD. Both diseases present cerebral hypoperfusion with associated degradation of BBB integrity which might impact the function of the glymphatic system. Neurovascular coupling is impaired in AD. Cerebral hypoperfusion is accompanied by reduced glucose metabolism which will transversely affect downstream pathways such as the ANLS. Astrocytic glycogen metabolism is regulated by noradrenaline and insulin being impaired in both diseases. Neuronal PPP is upregulated in AD and late PD as a response to increased oxidative stress. Oxidative stress is further exacerbated by disruption of the GSH flux from astrocytes to neurons. The transfer of neuronal peroxidated FA to astrocytes where they are degraded through FAO is impaired by the AD-related ApoE4 isoform, leading to the accumulation of such toxic FA. The Glu/GLn cycle is impaired in both conditions, due to defective removal of glutamate from the synaptic cleft which leads to excitotoxicity-induced neuronal loss. (Gluc, glucose; Glu, glutamate; Gln, glutamine; Lac, lactate; Pyr, pyruvate; Glyc, glycogen; R5P, ribose 5-phosphate; G6P, glucose 6-phosphate; GSH, glutathione; Cys,cysteine; Gly, glycine; FAO, fatty acid oxidation; TCA, tricarboxylic acid cycle). Figure created with images adapted from Servier Medical Art by Servier; licensed under a Creative Common Attribution 3.0 Unported License http://smart.servier.com/.

Despite accounting only for 2% of the body mass, the relative oxygen consumption of the adult human brain under resting conditions rises up to 20% of whole-body oxygen consumption (26, 27). Furthermore, when focusing on glucose oxidation rates in awake resting conditions, a figure that was broadly accepted, attributed 70-80% of those rates to neurons and approximately 20-30% to astrocytes. However, a re-examination of these proportions taking into account cell type volume fractions results in astrocytic glucose oxidation rates that exceed those of neurons (28). This assessment brought to light astrocytic energy demands that were not previously accounted for. In fact, the intricacies of the astrocyte-neuron metabolic dialogue are still under intense debate. Nevertheless, it is well appreciated that astrocytes play a central role in brain metabolic homeostasis, particularly concerning brain energetics and redox balance. Astrocytes serve as active regulators of their microenvironment and coordinate substrate availability with neuronal activity in a spatial-temporal manner. In the following sections, we discuss the most relevant biochemical processes that account for the astrocyte-neuron metabolic interplay. After a brief introduction of each process [for more details please refer to references (28, 29)], we will focus on known pathological mechanisms which disrupt the astrocyte-neuron dialogue in AD and PD (**Figure 2**).

### Cerebral Blood Flow and Neurovascular Coupling

The brain’s activity is sustained by a timely modulation of regional hemodynamics. The co-regulation of cerebral blood flow (CBF) and neuronal activity is known as neurovascular coupling (NC) or functional hyperemia and comprehends a complex set of regulatory steps performed by distinct cells including astrocytes (20). The molecular basis of NC is complex and results from a conjunction of feedback and feedforward mechanisms. While feedback regulation aims at supplying the target region with depleted energy substrates and removing by-products of intense neuronal activity (some of which have vasodilator properties), feedforward signaling is based on the release of vasoactive molecules [e.g. K^+^, prostanoids, nitric oxide (NO)]. The astrocytic participation in NC differs throughout the cerebrovascular tree, being more prevalent at the level of capillaries (20) and less important in arterioles (30). Astrocytes sense local increases in extracellular glutamate (the main excitatory transmitter) through metabotropic receptors resulting in the activation of a Ca^2+^-dependent signaling pathway, which will generate arachidonic acid and downstream vasodilating metabolites (such as prostaglandins and epoxyeicosatrienoic acids). Conversely, arachidonic acid itself can diffuse to vascular endothelial cells, where it can be transformed to vasoconstricting molecules such as 20-HETE (31, 32). These opposing roles of astrocyte activation on cerebral hemodynamics are dependent on NO levels, which regulate the routes of arachidonic acid conversion. The molecular orchestration regulating neurovascular coupling is much more complex and also includes adaptation to low O_2_ levels (20, 31).

Cerebral hypoperfusion caused by a combination of abnormalities in the cerebrovascular architecture as well as non-structural alterations was suggested as a prodromal feature of AD (30). BOLD fMRI studies investigating stimuli and task-triggered local cerebrovascular reactivity in AD patients and in cognitively normal individuals with an increased genetic risk for AD (APOE4 allele) revealed that neurovascular coupling is disrupted in preclinical stages of AD (32–34). Moreover, Aβ - the prime component of amyloid plaques - has vasculotoxic and vasoactive properties that mediate cerebrovascular deficiency (20, 30, 32).

Cerebral hemodynamics are also impacted in PD, with reduced CBF being reported in early stages of the disease (35). Moreover, in distinct regions of PD post-mortem brain tissue, degradation of vascular integrity was observed (20, 36). By contrast, several studies suggest that neurovascular coupling is sustained in PD (20, 37). Impaired nutrient input and accumulation of toxic metabolic sub-products due to cerebral hypoperfusion are worsened by the loss of BBB integrity, a well-recognized feature of early AD, which was also suggested to occur in PD (38–40). Furthermore, the disruption of BBB integrity is likely to enable the contamination of the brain parenchyma with blood-borne immunogenic molecules and to negatively impact the function of the glymphatic system, contributing in that manner to the build-up of toxic protein aggregates as seen in both pathologies (41, 42).

### Astrocyte-Neuron Energetic Coupling

Efficient exchange of energy substrates between the blood and the brain is sustained by the expression of distinct transporters, including GLUT1 for glucose, MCTs for monocarboxylates (such as lactate, pyruvate and ketone bodies) and FAT for fatty acids, at the level of the BBB (43). From these various molecules, glucose is the prime energetic substrate for the adult human brain, and its catabolism through primary (e.g. glycolysis and mitochondrial tricarboxylic acid (TCA) cycle) or secondary [e.g. pentose phosphate shunt pathway (PPP)] metabolic pathways not only generates ATP to fulfill the brain’s energy demands, but also provides critical precursors for the synthesis of neurotransmitters, neuromodulators and cellular components. In addition, glucose is important to sustain cellular antioxidant systems (28). Nevertheless, under specific circumstances, brain cells can rely upon other substrates that are imported from the blood (e.g. ketones bodies during starvation and lactate during intense activity) or produced locally (e.g. lactate, glutamine). Out of these alternative substrates, lactate was identified as a key player mediating the metabolic interplay between astrocytes and neurons. Both these cell types can efficiently metabolize glucose and lactate, however, while astrocytes present a more pronounced glycolytic profile, neurons preferentially rely on oxidative metabolism *via* mitochondrial oxidative phosphorylation (OXPHOS) (29). This notion was central to the formulation of the astrocyte-neuron lactate shuttle (ANLS) hypothesis (44), which was supported by studies suggesting incomplete glucose oxidation and/or increased lactate production following neuronal activity (26, 45).

#### Astrocyte-Neuron Lactate Shuttle (ANLS)

This model, which was first introduced in the early nineties (44), postulates that extracellular glutamate increase during intense neuronal activity leads to active astrocytic glutamate uptake, which in turn triggers Na^+^/K^+^ ATPase activation in astrocytes (to maintain Na^+^/K^+^ homeostasis) with an associated energy consumption and a drop in cellular ATP levels (46). To counteract this effect, astrocytic glucose uptake and glycolysis is elevated, secondarily increasing lactate production and excretion, which will then be available as a fuel to neuronal cells (47, 48). However, this concept is not consensual and evidence has been raised to dispute some of its premises (28). Interestingly, an alternative neuron-astrocyte lactate shuttle was also proposed (28).

Brain hypoperfusion is accompanied by reduced glucose and oxygen metabolic rates. This hypometabolism of glucose characterizes the normal aging process, with glucose metabolic rates decreasing by 26% from age 18 to 78 (49), but is further accentuated in pathological conditions such as AD and PD (38). In early AD, fluorodeoxyglucose-PET showed a characteristic reduction of total glucose metabolism in the parietotemporal association cortices, posterior cingulate cortex and the precuneus (49). Interestingly, aerobic glycolysis, which corresponds to the fraction of glycolysis not coupled to oxidative phosphorylation and which was previously associated with biosynthetic and neuroprotective roles, was found to be reduced in brain regions with higher levels of Tau deposition (50). Contrariwise, AD postmortem brain-derived data points to an overall upregulation of glycolytic enzymes, which was interpreted as a compensatory mechanism to mitochondrial dysfunction and to the reduced levels of glucose transporters that accompanies disease progression (51).

Alterations in glucose metabolism were also identified in brains from early- and late-stage PD patients (51). Several PD studies reported an extensive cortical hypometabolism of glucose, while only some of these investigations concomitantly show increased glucose metabolism in diverse subcortical regions (35, 52). Overall, disease-associated changes in glucose metabolism in both AD and PD are expected to profoundly disrupt brain cellular function, ultimately contributing to impaired neurotransmission, inefficient antioxidant defenses and neuronal death.

#### Glutamate/GABA-Glutamine Cycle

Astrocytes participate in the fast removal of neurotransmitters from the synaptic cleft, which is important for efficient signal termination (53). In the case of glutamate, this fast removal warrants the prevention of excitotoxicity. Astrocytes take up extracellular glutamate through high affinity sodium-dependent glutamate transporters (EAAT2) and the glutamate aspartate transporter (EAAT1). Astrocytic recycling of glutamate is instrumental to restore neuronal pools of this neurotransmitter, since neurons are deprived of key enzymes for glutamate *de novo* synthesis (53). Astrocytic glutamate can be converted to glutamine by the astrocyte-exclusive glutamine synthetase (54). Non-neuroactive glutamine is then safely redirected to neurons, where it can be converted back to glutamate through phosphate-activated glutaminase activity (53). Similar to this process, but at the level of GABAergic synapses, astrocytes take up part of the released GABA, which is processed through the TCA cycle in mitochondria. Glutamine released by astrocytes can equally be recovered by inhibitory GABAergic neurons to be used in the synthesis of GABA (53). Of note, both neurons and astrocytes can divert glutamate and glutamine for other uses such as oxidation in the TCA cycle or release from the brain for the maintenance of nitrogen balance across the BBB (29, 55). This net removal is compensated for *via* anaplerosis through the astrocyte-specific pyruvate carboxylase enzyme (55).

The previously mentioned hypometabolism of glucose occurring in AD and PD has a transversal effect on brain metabolism not only affecting directly communicating pathways such as the PPP, but also impairing downstream mechanisms such as the Glutamate/GABA-Glutamine cycle (56). Furthermore, neuronal loss in AD was linked to dysregulation of the glutamatergic system. A process that involves excitotoxicity, due to impaired glutamate uptake from the synaptic cleft. This impaired glutamate removal was further linked to lower activation of astrocytic glutamate transporters secondary to Aβ action (57, 58). Extensive evidence from distinct acute and genetic PD models identified an analogous glutamatergic dysfunction in this condition. Similarly, a defective glutamate uptake by astrocytes is instrumental for neuronal demise caused by glutamate excitotoxicity (59).

#### Astrocyte-Neuron Fatty Acid Coupling

Recently, an elegant mechanism coupling fatty acids (FA) metabolism in neurons and astrocytes was exposed (60). Extended periods of enhanced neuronal activity result in augmented generation of reactive oxygen species (ROS) and consequential accumulation of peroxidated FA (pFA). This poses an oxidative risk to the cell, since neurons have limited capacity to isolate those toxic species within lipid droplets. Instead, neurons release these pFA to the extracellular milieu in the form of ApoE-positive lipid particles, which are endocytosed by neighboring astrocytes and incorporated in lipid droplets. As a response to increased neuronal activity, astrocytes upregulate the expression of detoxification genes and initiate the breakdown of the lipid droplets, further catabolizing the released pFA to fuel oxidative phosphorylation in the mitochondria. Remarkably, this entire protective coupling mechanism is ApoE-isoform-dependent, and was shown to be seriously disrupted in mice knocked-in for the AD-relevant ApoE4 variant. This model presented compromised FA sequestration in neurons and decreased mobilization and energetic degradation in astrocytes (61). Interestingly, altered lipid metabolism has also been reported in PD, for instance in astrocytes derived from α-SYN KO mice (62).

#### Glycogen Storage

The astrocytic compartment is the most prominent site of glycogen storage in the brain, with accumulation of these energetic reserves being minute or inexistent in neurons (63). Interestingly, neuronal activity was shown to regulate astrocytic glycogen metabolism even in the presence of adequate glucose supply, disproving the idea that glycogen serves uniquely as an emergency energy reserve (29, 63). Further investigations are required to unravel the metabolic fates engaged in glycogen mobilization under distinct conditions. However, diversion of glycogen-derived glucose-6-phosphate into pathways such as the PPP have been observed under oxidative stress (64). Glycogen-derived lactate released by astrocytes is taken up by neurons and operates in the mechanism of long term memory consolidation (65) - a process that is hindered in AD. Indeed, decreased glycogenesis due to the inhibition of glycogen synthase was linked to overactivation of glycogen synthase kinase 3 (GSK-3) in AD (66). Furthermore, the dynamics of glycogen metabolism are regulated by factors such as noradrenaline (which stimulates the mobilization of glycogen stores, i.e. glycogenolysis) and insulin (which promotes glycogenesis) (26). Therefore, the degeneration of the noradrenergic system characteristic of AD (67) and the unbalanced insulin signaling occurring in AD and PD (26) will likely further contribute to the metabolic impairment found in these conditions.

#### Antioxidant System

The elevated cerebral metabolic rate along with an environment rich in unsaturated fatty acids and iron content and an inefficient antioxidant system renders the brain highly susceptible to oxidative stress (68). Also at this level, the astrocyte-neuron metabolic dialogue reveals its importance, since astrocytes, which are endowed with an intrinsically competent antioxidant system, convey some of this antioxidant potential to neurons (29). A good example of this is the shuttling of glutathione (GSH) precursors, the most prevalent antioxidant molecule in the brain, from astrocytes to neurons. Both cell types are capable of producing GSH, which might act as an independent ROS scavenger or as a substrate for antioxidant enzymes. However, neuronal cells strictly depend on astrocyte-derived GSH. Neurons express minimal levels of the System xc- cystine/glutamate antiporter, rendering them unable to efficiently capture cystine from the extracellular milieu. Cystine is the oxidized form of cysteine and is indispensable for the synthesis of GSH (68). Thus, neurons are dependent on the release of GSH by astrocytes. At the plasma membrane, GSH is converted by enzymes into glycine and cysteine, which are readily usable by neurons (29). The significance of this shuttling process is highlighted by the fact that neuronal GSH levels are dramatically increased when they are co-cultured with astrocytes (69).

Oxidative and nitrosative stress contributes to the pathogenesis of PD and AD (70), with robust evidence showing augmented oxidative stress markers in postmortem brain samples (71). Studies in PD patients showed significantly reduced glutathione levels in the substantia nigra (72), increased lipid peroxidation in the plasma and decreased activity of antioxidant enzymes (73). Moreover, the accumulation of Aβ in early AD results in increased oxidative stress (74). Under physiological and acute stress, astrocytes mediate neuroprotection by detoxifying the microenvironment from ROS and reactive nitrogen species (RNS), while providing neurons with precursors for glutathione synthesis. An example of a neuroprotective action performed upon astrogliosis is found in PD patients, in which reactive astrocytes elevate DJ-1 expression (75, 76). However, chronic reactive astrogliosis renders astrocytes neurotoxic, namely by the persistent production of ROS/RNS and release of immunogenic molecules (as discussed below) (70). Remarkably, disruption of normal brain metabolism can fuel this imbalanced oxidative status, since diminished levels of key PPP enzymes have been identified in the putamen of early-stage PD patients (71). This decreased flux through the PPP impairs the production of nicotinamide adenine dinucleotide phosphate (NADPH), which is a mandatory substrate for the regeneration of GSH pools by glutathione reductase. The decreased PPP flux was interpreted as a contributor to oxidative stress in early PD, while in later stages of the disease, the PPP flux would be upregulated in an attempt to counteract sustained oxidative stress (71). Interestingly, an increased PPP flux has been widely documented in AD as a metabolic adaptation to oxidative conditions (77). Furthermore, a recent mouse study showed that physiological ROS levels in astrocytes are needed to regulate metabolic rewiring, which modulates the PPP flux with potential implications on neuronal survival and cognitive impairment. This mechanism engaged the function of nuclear factor E2-related factor 2 (NRF2) and consequently, contributed to prevent disproportionate ROS release outside of the cell (78).

Moreover, a recent study associates PD with the hypermethylation and consequent downregulation in blood cells of *SLC7A11*, the gene coding for the System xc- cystine/glutamate antiporter. If maintained at the brain level, this dysregulation might account for a disruption of glutathione metabolism and glutamate neurotransmission (79).

Further examples of the causal relation between augmented oxidative stress and metabolic alterations in neurodegeneration can be found in the mitochondrial TCA cycle, were the activity of enzymes such as aconitase (80) and the α-ketoglutarate dehydrogenase complex (KGDHC) are impacted by reactive oxygen species (81). Paradoxically, these and other enzymes equally contribute to the production of ROS, further unbalancing the cellular redox status and aggravating disease mechanisms. KGDHC is a heteromeric enzyme that catalyzes the conversion of α-ketoglutarate to succinyl-CoA in a process that generates NADH and therefore supports ATP generation *via* OXPHOS. The regulatory process behind KGDHC activity is intricate and involves ADP/ATP, NAD^+^/NADH ratios, succinyl-CoA, Ca^2+^ and ROS levels (82). α-ketoglutarate is a metabolic precursor in the synthesis of glutamate, glutamine and GABA in diverse cell types, therefore the large scope of KGDHC functions, make this complex a leading molecular player integrating diverse cellular functions such as energy production, neurotransmission and redox homeostasis (83). Remarkably, changes in the activity of brain KGDHC have been reported in multiple neurodegenerative conditions, including PD (84). Moreover, such changes were extensively detailed in AD, where reduced KGDHC activity correlates with declined cognition (85).

Noteworthy, attempts to enhance defense mechanisms by specifically increasing the activity of ROS-sensing NRF2 has proven to be an efficient approach to maintain neuronal viability in both AD and PD models (74, 77, 86). NRF2 is a transcription factor primarily active in astrocytes, where it orchestrates cellular antioxidant responses and remodeling of intermediary metabolism. This example stresses the therapeutic potential that interventions targeting the astrocyte-neuron metabolic interaction have in the context of neurodegeneration (78).

#### Mitochondria and Astrocyte-Neuron Coupling

As previously mentioned, astrocytes and neurons fundamentally differ in their dependence on the mitochondrial oxidative phosphorylation. While ATP in astrocytes is mostly produced *via* aerobic glycolysis, they still possess functional mitochondria. However, the composition and organization of OXPHOS complexes in astrocytes is strikingly distinct from the respiratory chain structure in neurons. Initially, variations in complex I activity were observed when analyzing mitochondria in astrocytes and neurons from multiple species (87). Furthermore, it was shown that astrocytes contain a smaller percentage of complex I assembled into supercomplexes. These changes were correlated with an increase in ROS production in astrocytes (88). Overall, differences in the respiratory chain complex composition between astrocytic and neuronal cells, together with distinctive transcriptional and molecular regulation of key metabolic enzymes and substrate transporters (29), might explain higher glycolysis rates in astrocytes and their well-developed antioxidant defense system (88).

Multiple lines of evidence implicate PD-associated proteins in the regulation of mitochondrial function. One of the most prominent examples is α-SYN, which is relevant for both familial and idiopathic forms of PD. α-SYN effects on neuronal function can be either of primary or secondary nature. When localized to the mitochondria, the protein was shown to interfere with mitochondrial bioenergetics and biogenesis (89). By contrast, some studies also suggest that mitochondrial impairment can occur prior to α-SYN aggregation and potentially contribute to this phenomenon [reviewed in (89)]. Although predominantly expressed in neurons, α-SYN can be engulfed by astrocytes and affect their function. Notably, it was reported that such an uptake was more efficient for astrocytes than neurons, as demonstrated in murine co-cultures of astrocytes and neurons. Following the exposure to α-SYN, alterations in mitochondrial morphology and increased cell death were observed in the co-cultured neurons. Interestingly, neuronal death did not occur directly after the treatment, suggesting a neuronal response to the astrocytic dysfunction, presumably mediated by the release of astrocytic cytokines. Astrocytes could still survive in culture several days after exposure, underlining the importance of glycolysis for maintaining their function and neuronal dependence from undisturbed astrocytic metabolism (90). α-SYN is not the only PD protein, which may have a role in regulating mitochondrial processes in neurons as well as astrocytes. The relevance of PINK1 for mitochondrial homeostasis was explored in astrocytes from *PINK1*-knockout mice, which exhibited severe mitochondrial impairments (91). Another example is Parkin, which, when depleted in mice, caused stronger mitochondrial phenotypes in astrocytes than in neurons. Furthermore, Parkin-deficient astrocytes were not able to promote neuronal differentiation *in vitro* (92). Additionally, the PD protein DJ-1, which is highly abundant in astrocytes and involved in the oxidative stress defense, was shown to play a key role in maintaining proper mitochondrial function in astrocytes (93). In the absence of DJ-1, these cells lose their ability to protect neighboring neurons from oxidative stress (94). Since data on the role of astrocytic mitochondria in PD is still sparse [reviewed in (76, 95)] further experiments will be required to assess how astrocyte metabolism as well as astrocyte-neuron coupling may contribute to the development of the movement disorder.

Similar to the scenario described above for α-SYN in PD, Aβ has been shown to affect mitochondrial function and metabolism in AD neurons. Thus, some authors argue that mitochondrial impairment precedes Aβ pathology in AD (95). Overall, the available mitochondrial function data in astrocytes from AD mouse or patient models is very limited (96). Therefore, more studies will be required to shed light on possible effects of this dysfunction on astrocyte-neuron coupling.

#### Astrocytic Calcium Signaling and Astrocyte-Neuron Crosstalk

Interestingly, mitochondria were shown to be involved in intracellular calcium signaling in astrocytes (97). Calcium release from mitochondria can trigger spontaneous Ca^2+^ oscillations in astrocytic processes - a phenomenon attributed to the opening of the mitochondrial permeability transition pore when oxidative phosphorylation is particularly active. This suggests a direct connection between metabolic demand and calcium signaling (98). Spontaneous Ca^2+^ waves can be propagated to other astrocytes and ultimately cause neuronal activity. Furthermore, it was proposed that the described process might play an important role in brain development, by supporting the formation of synaptic connections and the regulation of the neurotransmission (99).

Although astrocytes have been considered passive bystanders in neurotransmission events, this view was recently challenged. It became clear that astrocytes are able to respond to neurotransmitters as manifested by changes in Ca^2+^ levels. Based on this response, astrocytes were shown to react to glutamate, acetylcholine, ATP, GABA, and endocannabinoids. In return, they are able to release glutamate, D-serine, ATP, and GABA influencing activity of the neighboring neurons (100).

Alterations in Ca^2+^ signaling were identified in reactive astrocytes in the context of neurodegeneration. Generally, these changes were characterized by an enhanced amplitude, duration and frequency of these signals (97). In an AD mouse model, it was shown that astrocytes surrounding Aβ plaques might generate aberrant Ca^2+^ signals, which can be further propagated to other astrocytes (101, 102). It was hypothesized that changes in Ca^2+^ signaling might be one of the mechanisms, which play a role in the contribution of reactive astrocytes to the pathology of neurodegenerative diseases, however, this possibility requires further examination (97).

As discussed in the previous sections, neuronal activity marked by glutamate release can elicit metabolic changes in astrocytes as evidenced by considerably higher glucose uptake (103). Recently it became clear that such metabolic modulation is elicited by dual Na^+^ and Ca^2+^ signaling, which triggers glucose mobilization and subsequent aerobic glycolysis to support neuronal functions by providing lactate. In contrast, spontaneous calcium spikes in astrocytes do not trigger a similar response, suggesting that astrocytic metabolism is mainly focused on meeting high metabolic demands of neurons (104, 105).

## iPSC-Derived Astrocyte Models to Study Metabolism in Neurodegenerative Diseases

In recent years, we have witnessed major breakthroughs in modeling human diseases after the iPSC technology was implemented. Since then, we observed the development of numerous protocols for the generation of iPSC-derived astrocytes (106–110) [for an extensive review of earlier protocols see (111)], allowing researchers to shed light on the involvement of astroglia in the pathogenesis of human neurodegenerative diseases. Such tools proved to be also useful in studying metabolic alterations in the brain (**Table 1**), in particular taking into account the above-mentioned contribution of astrocytic metabolism to the maintenance of neuronal function. In astrocytes derived from AD patients harboring *PSEN1* mutations, fatty acid oxidation was compromised (112), in line with earlier reports showing decreased levels of fatty acid oxidation products in AD patients (122) and the studies regarding the astrocyte-neuron FA-coupling mentioned above. Importantly, Konttinen and colleagues identified a plausible target for AD treatment, since the fatty acid oxidation impairment was rescued with a compound (GW0742), which triggered the activation of PPARβ/δ, a ligand-inducible transcription factor controlling lipid and glucose metabolism (112, 123). Another variant linked to AD, *APOE4*, was shown to cause altered expression of various lipid metabolism genes in iPSC-derived astrocytes. Furthermore, the intra- and extracellular levels of cholesterol were increased in these cultures (115). Changes in cholesterol levels were also observed in another study using iPSC-derived astrocytes harboring the Swedish mutation in the amyloid precursor protein (APP) gene (117). These findings suggest that cholesterol metabolism might be implicated in the pathogenesis of AD.

**Table 1:** The summary of patient-derived models used to elucidate the effects of neurodegeneration-linked mutations on astrocytic metabolism.

Disease	Study	Gene	Mutation/variant	Type of cells	Main finding	Astrocytic protocol used	Comments
AD	Konttinen et al. (112)	*PSEN1*	Deletion of exon 9	Astrocytes, murine neuroprogenitor cells	Impaired fatty acid oxidation, rescued by GW0742 treatment	Krencik et al. (113); modified in Oksanen et al. (114)	Isogenic controls included
AD	Lin et al. (115)	*APOE*	*APOE4, APOE3*	Astrocytes, neurons, microglia, organoids	Increased levels of cholesterol and reduced Aβ uptake in *APOE4* astrocytes	Chen et al. (116)	Isogenic controls included
AD	Fong et al. (117)	*APP*	KO, V717F, Swedish	Astrocytes, neurons, neuroprogenitor cells	Decreased lipoprotein endocytosis and increased SREBP levels in APP-KO astrocytes, accompanied by reduced Aβ uptake, astrocytes harboring Swedish mutation mimic this phenotype	Yuan et al. (118); modified in the study	Isogenic controls included
PD	Sonninen et al. (119)	LRRK2, GBA	LRRK2 G2019S, GBA N370S	Astrocytes	Increased α-SYN levels, changes in metabolism, particularly in polyamines and lysophosphatidylethanolamine levels, altered calcium signaling	Krencik et al. (113); modified in Oksanen et al. (114)	Isogenic controls included
FTD	Aldana et al. (120)	*CHMP2B*	H150, H151, H242	Neurons, astrocytes	Altered glutamine-glutamate related pathways in neurons, in astrocytes enhanced glutamate uptake	Shaltouki et al. (121); modified in the study	Isogenic controls included

AD, Alzheimer’s disease; PD, Parkinson’s disease; FTD, frontotemporal dementia.

In the context of PD, several metabolic changes were detected in astrocytes derived from patients harboring *LRRK2* mutations. The secretion to the medium was increased for several polyamines, such as putrescine and spermidine and the levels of their precursors, arginine and ornithine, were elevated in astrocytes. Polyamines are important for various cellular processes, such as cell proliferation and differentiation, gene transcription and translation, as well as regulation of ion channel and receptor activity (119). Notably, changes in polyamine levels were also detected in the cerebrospinal fluid (CSF) of PD patients supporting the relevance of the data obtained in the iPSC models (124). Moreover, in the same study from Sonninen et al., a decline in phospholipids levels, in particular lysophosphatidylethanolamine, was observed in PD astrocytes, which is again in agreement with data obtained from the analysis of CSF from PD patients (119, 125). Another important aspect of astrocytic metabolism, namely glutamine uptake and conversion, was studied in the context of frontotemporal dementia. In this case iPSC-derived astrocytes presented substantial changes in glutamate uptake (120).

Although mainly implemented to study metabolic changes in human diseases, iPSC models of astrocytes proved their uttermost importance in elucidating molecular pathways governing metabolism of valine and medium-chain fatty acids in the brain. These findings might be useful to determine an optimal ketogenic diet, which was shown to have a positive impact on the health of patients suffering from traumatic brain injury, glucose transporter I deficiency, AD and epileptic seizures (126–128).

## Astrocytes and Mitochondria in Inflammation

As touched on in the third section of this review, astrocytes are immunocompetent cells. Accordingly, stimulation with interferon (IFN) γ and/or tumor necrosis factor (TNF) α can trigger the expression of major histocompatibility complex (MHC) class II molecules, which are required for antigen presentation (129). Moreover, *in vitro* studies in human immortalized astrocytes and primary mouse astrocytes have shown that, in response to IFN-γ, astrocytes are able to activate naïve T cells. However, there is conflicting evidence as to whether class II MHC+ astrocytes can stimulate the proliferation of activated T-helper (Th) type 1 or 2 cells (129).

**Figure 3:**
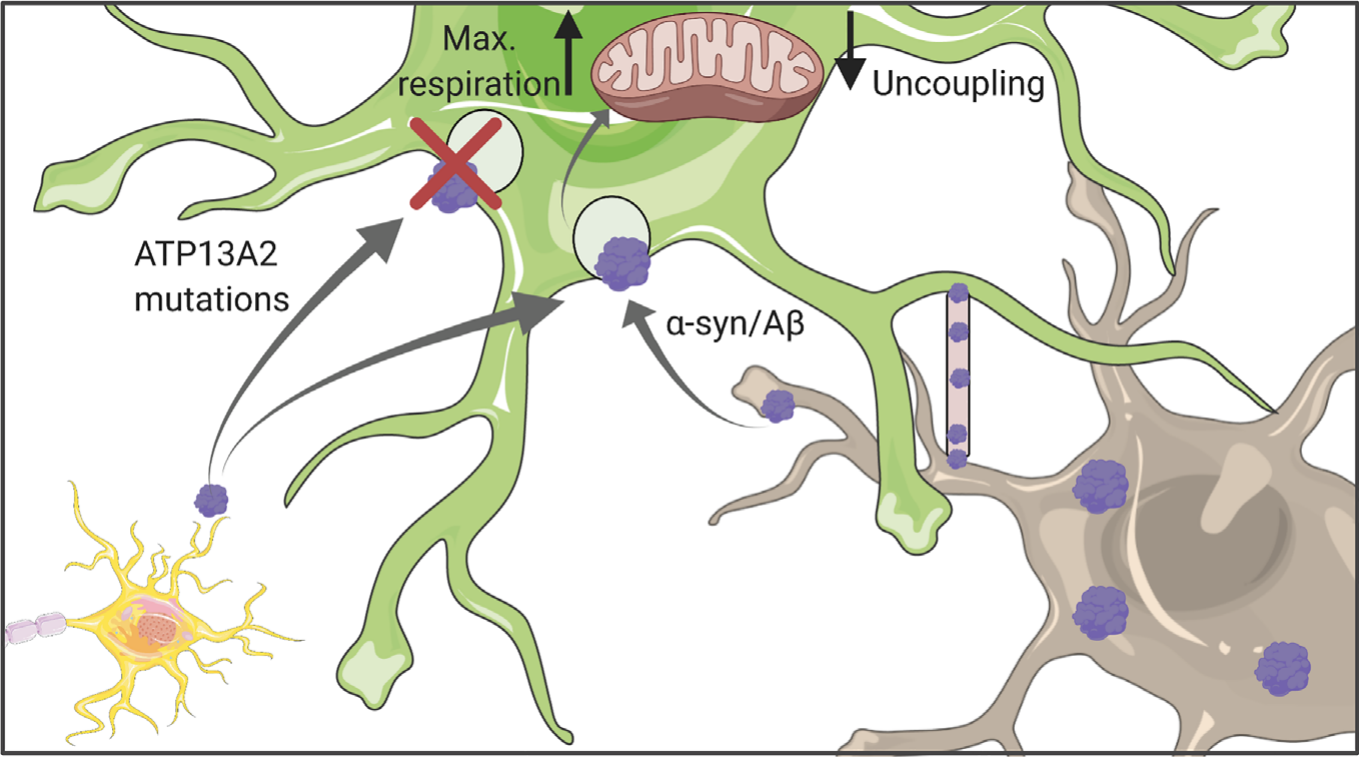
Astrocytic uptake of protein aggregates. Astrocytes can internalize both Aβ and α-synuclein which might originate either from other astrocytes or neurons. Uptaken aggregates were shown to affect mitochondrial function, in particular their respiration capacity and coupling status. Figure created with BioRender.com using images adapted from Servier Medical Art by Servier, licensed under a Creative Common Attribution 3.0 Unported License http://smart.servier.com/.

While the antigen-presenting properties of astrocytes are still under debate, there are a myriad of studies (130) highlighting that astrocytes can release cytokines including IL-1, -6 and -10; TNF-α, transforming growth factor (TGF) β as well as IFN-α and -β in response to molecular triggers (129). Such signals can be of extra- or intracellular nature and are detected by pattern recognition receptors (PRR). Contrary to acute injury models, in neurodegenerative disease it is suspected that mild but progressive cues trigger astrocyte reactivity. Situated at the tripartite synapse, astrocytes have the capacity to sense altered neurotransmission patterns and neuronal stress signals (131). As previously mentioned, astrocytes can engulf neurotoxic proteins such as α-SYN (132) and Aβ (133). These aggregates can then be transferred from diseased to healthy cells *via* tunneling nanotubes (134) (**Figure 3**). Moreover, experiments with iPSC-derived astrocyte-neuron co-cultures revealed that astrocytes rapidly internalize neuronal α-SYN. By contrast, this intercellular transfer was prohibited in cultures from PD patients with mutations in the lysosomal storage protein ATP13A2 (135). This data not only suggests that astrocytes contribute to the neuronal α-SYN pathology in genetic PD, but that they may also act as mediators in neuroinflammatory processes. In line with this hypothesis, selective overexpression of A53T-mutant α-SYN in murine astrocytes induced rapidly progressing paralysis, which was the result of an overshooting inflammatory response. Astrocytic expression of A53T α-SYN triggered microglial activation and neurodegeneration in this PD mouse model (136). As discussed in sub-section *Mitochondria and Astrocyte-Neuron Coupling*, α-SYN likely interferes with astroglial and neuronal mitochondria. Thus, the proinflammatory action of α-SYN may be amplified by mitochondrial impairment in both cell types. In fact, in mice, depletion of the mitochondrial transcription factor A (TFAM), which controls transcription, replication and 3D structure of the mitochondrial genome, caused the activation of the cGAS-STING inflammatory pathway. With regard to PD, our own research revealed that, in post-mortem nigral dopaminergic neurons from sporadic patients with α-SYN pathology, TFAM deficiency is associated with reduced respiratory chain complex I protein levels (137). In the absence of TFAM, mitochondrial DNA (mtDNA) is released into the cytosol and the extracellular space, where it acts as damage-associated molecular pattern (DAMP) (138). As mitochondrial DAMP, mtDNA does not only have the capacity to initiate cGAS-STING signaling, but also to activate the NLRP3 inflammasome. In fact, CMPK2 - the rate-limiting enzyme supplying deoxyribonucleotides for mtDNA synthesis - is required for the generation of oxidized mtDNA fragments that are recognized by the NLRP3 complex in the cytosol (139). Supporting these observations, single-cell RNA sequencing and quantitative immunofluorescence analyses of control and IPD midbrain sections indicated an upregulation and activation of astroglial cells in the patient tissue (140). To test whether α-SYN accumulation, mitochondrial dysfunction, neurodegeneration and astroglial immune response are indeed causally linked in PD, iPSC-derived (co-culture) models will be useful. First experiments in astrocytes derived from healthy controls suggest that treatment with high-molecular-weight α-SYN fibrils reduces the secretion of pro-inflammatory cytokines. Similar to the situation in neurons, mitochondrial respiration in these cells was impaired in response to α-SYN exposure. This effect was even more pronounced in PD patient astrocytes harboring *PRKN* mutations (141).

Beyond DAMPs of neuronal (or even astroglial) origin, molecules released from microglia can initiate signaling cascades that mediate astrocyte reactivity (131). Liddelow and colleagues identified two classes of reactive astrocytes, which they termed A1 and A2 in analogy to the nomenclature of macrophages. While A1 astrocytes act neurotoxically, A2 astrocytes have neuroprotective function (25). A1 astrocytes are activated by cytokines such as Il-1α, TNF or C1q secreted from microglia. As a consequence, they promote neuronal death by hampering outgrowth, synaptogenesis and phagocytosis. Consistent with these properties, A1 astrocyte numbers are elevated in various neurodegenerative disorders including PD (24). Interestingly, mitochondria (as a source of DAMPs) are not only involved in the initiation of inflammation (142), they are also crucial for the modulation of the inflammatory response. Experiments in mouse cortical astrocytes suggest a transient change in mitochondrial dynamics coinciding with elevated ROS levels and autophagy induction in response to proinflammatory stimuli (143). This spontaneous upregulation of mitochondrial clearance ensures astrocyte survival and may, in turn, influence neuronal metabolism (143).

## Discussion

Taken together, there is increasing evidence that mitochondrial dysfunction and disrupted metabolism impact on astrocyte-neuron coupling in AD as well as PD. Moreover, a multitude of studies indicate that the oxidative stress defense system is impaired in both disorders. By contrast, the sequence of events causing these phenotypes currently remains elusive. Further experimental work will be required to clarify whether (i) primary mitochondrial dysfunction due to mutations in (one or multiple) proteins relevant for mitochondrial homeostasis could lead to oxidative stress and metabolic disruptions or whether (ii) protein aggregation and metabolic imbalances cause secondary mitochondrial dysfunction thereby aggravating neuronal pathology. Ultimately, faulty mitochondria may release DAMPs that could trigger inflammation - a less explored aspect of astroglial metabolism and function. Equally, it should be noted that, to date, very few metabolic findings from mouse models have been validated in human astrocytes. By contrast, various protocols for the generation of pure astrocyte cultures from iPSCs have been published in the recent years. In-depth characterization experiments indicate that these cultures may not only serve as models to study disease mechanisms but that they could be used for drug-screening approaches in the future. By combining mutant neurons with isogenic control astrocytes and vice versa, we may be able to better define the predominant metabolic role and cell type of action for proteins associated with AD or PD. Thus, patient-derived astrocyte-neuron co-culture systems hold great potential for the exploration of metabolic modulation strategies in neurodegenerative disorders.

## Author Contributions

PM, AG, and SP designed the structure of the review. PM, AG, and SP performed literature search, wrote the draft and edited the manuscript. The figures were prepared by PM and SP. All authors contributed to the article and approved the submitted version.

## Funding

PM, SP, and AG were supported by the Luxembourg National Research Fund (FNR) within the CORE (CAMeSyn, C19/BM/13688526) and ATTRACT programs (Model-IPD, FNR9631103). In addition, PM was supported by the FNR *via* the PARK-QC DTU (PRIDE17/12244779/PARK-QC). AG and SP were supported by a donation from Le Foyer Assurances Luxembourg, which was matched by the FNR within the framework of the PARK-QC DTU program.

## Conflict of Interest

The authors declare that the research was conducted in the absence of any commercial or financial relationships that could be construed as a potential conflict of interest.
